# Fast Inverse Distance Weighting-Based Spatiotemporal Interpolation: A Web-Based Application of Interpolating Daily Fine Particulate Matter PM_2.5_ in the Contiguous U.S. Using Parallel Programming and k-d Tree

**DOI:** 10.3390/ijerph110909101

**Published:** 2014-09-03

**Authors:** Lixin Li, Travis Losser, Charles Yorke, Reinhard Piltner

**Affiliations:** 1 Department of Computer Sciences, Georgia Southern University, Statesboro, GA 30460, USA; E-Mail: tlosser1@gmail.com; 2 Department of Geosciences, Murray State University, Murray, KY 42071, USA; E-Mail: cyorke@murraystate.edu; 3 Department of Mathematical Sciences, Georgia Southern University, Statesboro, GA 30460, USA; E-Mail: rpiltner@georgiasouthern.edu

**Keywords:** fine particulate matter PM_2.5_, spatiotemporal interpolation, inverse distance weighting (IDW), parallel programming, k-d tree, leave-one-out cross-validation, k-fold cross validation, web-based application, visualization

## Abstract

Epidemiological studies have identified associations between mortality and changes in concentration of particulate matter. These studies have highlighted the public concerns about health effects of particulate air pollution. Modeling fine particulate matter PM_2.5_ exposure risk and monitoring day-to-day changes in PM_2.5_ concentration is a critical step for understanding the pollution problem and embarking on the necessary remedy. This research designs, implements and compares two inverse distance weighting (IDW)-based spatiotemporal interpolation methods, in order to assess the trend of daily PM_2.5_ concentration for the contiguous United States over the year of 2009, at both the census block group level and county level. Traditionally, when handling spatiotemporal interpolation, researchers tend to treat space and time separately and reduce the spatiotemporal interpolation problems to a sequence of snapshots of spatial interpolations. In this paper, PM_2.5_ data interpolation is conducted in the continuous space-time domain by integrating space and time simultaneously, using the so-called extension approach. Time values are calculated with the help of a factor under the assumption that spatial and temporal dimensions are equally important when interpolating a continuous changing phenomenon in the space-time domain. Various IDW-based spatiotemporal interpolation methods with different parameter configurations are evaluated by cross-validation. In addition, this study explores computational issues (computer processing speed) faced during implementation of spatiotemporal interpolation for huge data sets. Parallel programming techniques and an advanced data structure, named k-d tree, are adapted in this paper to address the computational challenges. Significant computational improvement has been achieved. Finally, a web-based spatiotemporal IDW-based interpolation application is designed and implemented where users can visualize and animate spatiotemporal interpolation results.

## 1. Introduction

### 1.1. Background

Since the beginning of the nineteenth century, the human population has been increasing at an alarming rate. As population increases, so do human needs and demands for various resources for their survival. Currently, demand for food, potable water, clean air and energy, as well as the demand for habitable land are increasing. In the same direction, population increase requires more land for liquid and solid waste disposal [1]. However, the need for more land for liquid and solid waste disposal leads to an increase in the amounts of pollutants in our environment that affect the health of more and more people, including elderly and children [2,3,4]. The health effects of pollutants have been subject to intense study in recent years. This paper focuses on monitoring the trend of daily air pollution using fine particulate air pollutant (PM_2.5_) concentration in the contiguous United States.

Epidemiological studies have identified certain associations between mortality and changes in concentration of particulate matter [5,6,7,8]. These studies have highlighted the public concerns about health effects of particulate air pollution. Particulate air pollution is a mixture of liquid droplets and solid particles that varies in origin, size and composition. PM_2.5_ is considered a fine particle, not an ultra-fine particle. Ultra-fine is a term reserved for particulate matter smaller than 0.1 micron (100 nanometers) in size; particles that are on a nano-scale. The EPA sets standards for the maximum amount of PM_2.5_ that can be in the ambient outdoor air with the goal of protecting health. Cities and states must then comply with these standards. However, the EPA has not set any standards for the smaller, ultra-fine particles. PM_2.5_ contains particles with an aerodynamic diameter of 2.5 micrometers or below. PM_2.5_ typically contains a mixture of particles, such as acid condensates, soot and sulfate and nitrate particles. PM_2.5_ is thought to pose a particularly great risk to people’s health, because it is more likely to be toxic and can be breathed more deeply into the lungs.

To establish associations between pollutants concentration and health effects, researchers have relied on exposure assessment models to estimate exposure risk to pollutant [1,9,10,11,12,13], since pollutants measurements occur at certain point locations. Currently, several groups of exposure risk assessment models have been developed, including geostatistical models [14,15], proximity models [16,17], air dispersion models [18,19] and deterministic models [20]. This paper focuses on one of the deterministic models, called IDW (inverse distance weighting) interpolation. Some of the advantages for the IDW interpolation method include:

- IDW interpolation is simple and intuitive.
- IDW interpolation is fast to compute the interpolated values.

Some of the disadvantages the IDW interpolation method include:

- The choice of IDW interpolation parameters are empirical (*i.e*., based on, concerned with or verifiable by observation or experience rather than theory or pure logic).
- The IDW interpolation is always exact (*i.e*., no smoothing).
- The IDW interpolation has sensitivity to outliers and sampling configuration (*i.e*., clustered and isolated points).

### 1.2. Literature Review on Interpolation in GIS

Since air pollution concentrations are typically measured at certain point locations and certain time instances by monitoring sites, estimation or prediction of pollutant concentrations at unmeasured locations and times is the foundation for research investigating the associations between pollutants and health effects. This procedure of estimation or prediction is called interpolation.

Spatial interpolation refers to the estimation of values at unsampled points based on known values of surrounding points in space. It is commonly used in a Geographic Information System (GIS) to generate a continuous layer of data from a set of point data taken at sample locations, in order to estimate elevation, rainfall, temperature, chemical dispersion, pollution or other spatially-based continuously changing phenomena. There are a number of spatial interpolation algorithms, such as IDW (inverse distance weighting) [21], Kriging [22], shape functions [23], spline [24] and trend surface [25]. All of the spatial interpolation methods assume a stronger correlation among points that are closer than those farther apart, which is known as Tobler’s First Law of Geography [26]. In summary, spatial interpolation methods are well developed and widely adopted in various GIS applications [27,28,29,30,31,32].

In the recent decade, spatiotemporal interpolation has gained attention in the GIS interpolation research community [33,34,35,36,37,38,39]. Spatiotemporal interpolation involves estimation of the unknown values at unsampled location-time pairs with a satisfying level of accuracy [40]. However, when applying traditional spatial interpolation methods for spatiotemporal data, researchers face many challenges. One of the major challenges is that traditional GIS researchers tend to treat space and time separately when interpolation needs to be conducted in the continuous space-time domain. The primary strategy identified from the literature is to reduce spatiotemporal interpolation problems to a sequence of snapshots of spatial interpolations [41]. However, integrating space and time simultaneously can yield better interpolation results than treating them separately for certain typical GIS applications [42].

In order to integrate space and time simultaneously for a spatiotemporal interpolation, an extension approach has been proposed in [40] and reviewed in [43,44]. This approach extends the spatiotemporal interpolation into a higher-dimensional spatial interpolation by treating time as another dimension in space. Therefore, the extension approach can be used with any spatial interpolation method that can be extended to higher dimensions. Some applications using the extension approach can be found in [40,45,46].

The two of the most commonly used spatial interpolation methods in GIS applications are IDW and Kriging [20,47,48,49,50]. Many studies have examined the relative performance of IDW and Kriging. In most cases, the findings have been mixed [51]. In some studies, it was found that the performance of IDW was not as good as Kriging [52]. However, in other studies, IDW outperformed Kriging [53].

Kriging and IDW interpolations are similar in that both weight the surrounding measured values to derive a prediction for an unmeasured location [54]. However, Kriging and IDW interpolations have one main difference. In Kriging, the weights are based not only on the distance between the measured points and the prediction location, but also on the overall spatial arrangement among the measured points [54]. Therefore, Kriging depends on a fitted model to the measured points. Whereas IDW is a deterministic interpolator that depends solely on the distance to the prediction location to interpolate the unmeasured location [54].

Several epidemiological studies have used IDW-based interpolation methods to assess population exposure to pollutants [55,56,57,58]. For example, Brauer, Lencar, Tamburic, Koehoorn, Demers and Karr [58] employed the IDW-based interpolation technique to analyze air pollution exposure for adverse effects on pregnancy outcomes by examining individual-level intra-urban exposure contrasts. Hoek, Fischer, Van Den Brandt, Goldbohm and Brunekreef [55] used the IDW-based interpolation technique to investigate the relationship between traffic-related air pollution and mortality. IDW-based interpolation methods have been used for their simplicity and efficiency.

In view of this background, we design and implement in this paper IDW-based spatiotemporal interpolation using the extension approach to assess the trend of daily PM_2.5_ concentrations for the contiguous United States in 2009.

## 2. Methods

### 2.1. Experimental PM_2.5_ Data

To assess the trend of air pollution over the course of a year in the contiguous United States, daily PM_2.5_ concentration is experimented on in this paper. Three sets of data were used.

**Figure 1:**
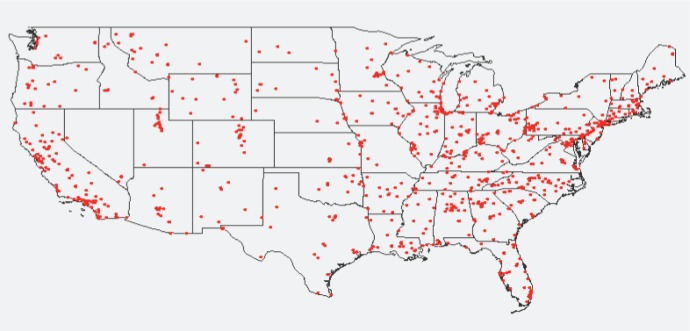
The 955 monitoring sites with particulate matter (PM_2.5_) measurements.

The first dataset stores PM_2.5_ measurements that were obtained from the U.S. Environmental Protection Agency (EPA). The data coverage contains point locations of the monitoring sites, the concentration measurements of PM_2.5_ and the days when the measurements were taken in 2009. In detail, the dataset contains 146,125 PM_2.5_ measurements collected at 955 monitoring sites on 365 days in 2009. It has the following attributes: id, year, month, day, x, y and PM_2.5_ concentration measurement, where x and y are the longitude and latitude coordinates of the monitoring sites. The PM_2.5_ concentration measurement’s unit is micrograms per cubic meter (*μg/m*^3^). The locations of the monitoring sites are illustrated as red dots in Figure 1.

The second and third datasets contain centroid locations of counties and census block groups in the contiguous U.S. More specifically, the second dataset contains the centroid locations of 3109 counties, and the third dataset contains the centroid locations of 207,630 census block groups. A census block group is a geographical unit used by the United States Census Bureau that is between the census tract and the census block. A census block group is generally defined to contain between 600 and 3000 people. It is the smallest geographical unit for which the bureau publishes sample data, that is, data which are only collected from a fraction of all households [59].

The goal of this paper is to develop and implement a fast IDW-based spatiotemporal interpolation method to estimate the daily PM_2.5_ concentration values in 2009 at the centroids of counties and census block groups (the second and third datasets) for the entire contiguous U.S., using the existing PM_2.5_ measurements (the first dataset) as input.

### 2.2. IDW-Based Spatiotemporal Interpolation Method Using the Extension Approach

In order to develop an efficient spatiotemporal interpolation method suitable for the daily PM_2.5_ data, the IDW-based spatiotemporal interpolation method using the extension approach that treats time as an equivalent to the spatial dimensions [40] is considered in this paper. The IDW method is a simple and intuitive deterministic interpolation method based on Tobler’s First Law of Geography [26] that assumes that sample values closer to the unmeasured location of interest have more influence on the interpolated value than sample values farther apart. Measured points that are closer to the unmeasured point are given a higher weight than measured points that are farther away. Thus, the IDW interpolation assumes that each measured point has a local influence that diminishes with distance and weighs the points closer to the interpolated location greater than those farther away.

#### 2.2.1. Original IDW-Based Spatiotemporal Interpolation Method Using the Extension Approach

We adapt the traditional spatial IDW method and utilize the following spatiotemporal interpolation formula based on the extension approach:

(1) $$w(x,\;y,\;ct)=\sum_{i=1}^{N}\lambda_{i}w_{i}\;\;\;\;\;\;\;\;\;\;\;\;\;\;\;\;\;\lambda_{i}=\frac{{\left(\frac{1}{d_{i}}\right)}^{p}}{\sum_{k=1}^{N}{\left(\frac{1}{d_{k}}\right)}^{p}}$$

where *c* is a factor with the unit [*spatial distance unit/time unit*], *w*(*x*, *y*, *ct*) is the interpolation value to calculate at the unmeasured location (*x*, *y*) and time instance *t*, *N* is the number of nearest neighbors with measured values surrounding (*x*, *y*, *ct*), *λ_i_* are the weights assigned to each measured value *w_i_* at (*x_i_*, *y_i_*, *ct_i_*), *d_i_* is the spatiotemporal Euclidean distance between (*x_i_*, *y_i_*, *ct_i_*) and (*x*, *y*, *ct*) and *p* is the exponent that influences the weighting of *w_i_*. Weighting value *λ_i_* ranges from zero to one and is a function of the inverse of the spatiotemporal distance between a nearest neighbor and the unmeasured point. It is worth noting that the sum of all of the *λ_i_*s(*i* ∈ [1, *N*]) is one. The spatiotemporal Euclidean distance *d_i_* is calculated using the following formula by getting the sum of three distances squared under the root sign:

(2) $$d_{i}=\sqrt{{\left(x_{i}-x\right)}^{2}+{\left(y_{i}-y\right)}^{2}+c^{2}{\left(t_{i}-t\right)}^{2}}.$$

Since time is treated as an equivalent to spatial dimensions using the extension approach, it is not difficult to see from Equations (1) and (2) that different factor *c* values will cause different values of *d_i_*, *λ_i_*, and eventually lead to different interpolation results of *w*. Therefore, the choice of the factor *c* is crucial and needs to be addressed, because it affects the IDW interpolation performance.

A naive way of choosing the time values for the experimental PM_2.5_ data is to use incremental values, such as incrementing one per day and considering the factor *c* as one, as shown in the second column in Table 1. However, it has been shown from previous work [42] that this naive choice for time values does not yield the best interpolation results.

**Table 1:** Two choices of time values: a naive choice of *t* and a scaled choice of *c* * *t* for the PM_2.5_ data set, where the factor *c* =0.1086.

**Day**	**t**	c * t
1 January 2009	1	0.1086
2 January 2009	2	0.2172
3 January 2009	3	0.3258
4 January 2009	4	0.4344
...	...	...
31 December 2009	365	39.6390

An idea has been proposed in [60] to scale time values for a spatiotemporal data set, so that the range in the time extent of an input dataset is equal to the average ranges of the spatial extents. This will lead to a factor *c* that is not equal to one. The assumption behind this idea is that spatial and temporal dimensions are equally important when interpolating a continuous changing data set in the space-time domain. Based on this idea, the factor *c* in Equations (1) and (2) for the PM_2.5_ data can be computed as:

(3) $$c=\frac{1}{364}*\frac{\left(x_{\max}-x_{\min})+(y_{\max}-y_{\min}\right)}{2}$$

where *x_max_* is the largest x coordinate, *x_min_* is the smallest x coordinate, *y_max_* is the largest y coordinate and *y_min_* is the smallest y coordinate. After plugging the actual *x_max_*, *x_min_*, *y_max_* and *y_min_* values from the PM_2.5_ dataset into Equation (3), we get *c* =0.1086, where actual *x_max_* = −68.0162, *x_min_* = −124.1775, *y_max_* =48.3997, *y_min_* =25.4714.

In this paper, we use the scaled time values *c* * *t* as shown in the third column in Table 1, where *t* is the naive choice of time using incremental values with one increment per day.

#### 2.2.2. Improved IDW-Based Spatiotemporal Interpolation Method Using the Extension Approach

The IDW-based spatiotemporal interpolation method using the extension approach is improved by excluding nearest neighbors that are far away from the interpolation point. Objects that are close to one another are more alike than those that are farther away. Therefore, as the neighbors get farther away, the measured values have little relationship to the value of the prediction point. Hence, it is better to exclude the more distant neighbors that have little influence on the interpolation.

Using the fixed number of nearest-neighbors approach will always find neighbors, but they may be so far away from the interpolation points that the IDW interpolation may be giving misleading information. Therefore, it is better to limit the number of measured values by specifying a search neighborhood. The search neighborhood restricts how far and where to look for the measured values to be used in the interpolation. The distance calculation uses both space and time when determining how far the neighbor is from the interpolation point.

The improved IDW-based interpolation uses attributes for the maximum Euclidean distance and the maximum time difference, such that points in sparser neighborhoods use fewer nearest neighbors in the interpolation calculation. The maximum for the Euclidean distance and the time difference are the maximum values that are allowed between the nearest neighbor and the point being interpolated. For example, if the date of the interpolation point is 20 April 2009, it does not make sense to use a measured value with a date of 20 December 2009, when calculating the interpolated value, even if the measured value was determined to be a nearest neighbor.

#### 2.2.3. Discussion of the Methods

In this paper, we use inverse distance functions *λ_i_*, which depend on distances *d_i_* and *d_k_* from Equation (1). The inverse distance function can be considered a special case of a radial basis function. Radial basis functions (RBFs) became quite popular in recent years. Good introductions of radial basis function methods are the books of Fasshauer [61] and Wendland [62].

Radial basis functions can be used for higher dimensional problems. They are not restricted to two- and three-dimensional spatial problems. We can use the following distance *r* for an n-dimensional problem:

(4) $$r=\sqrt{c_{1}^{2}x_{1}^{2}+c_{2}^{2}x_{2}^{2}+c_{3}^{2}x_{3}^{2}+\ldots c_{n}^{2}x_{n}^{2}}$$

The coefficients *c_i_* can be used for scaling purposes and to achieve the same physical unit for each term. In our examples, we use the following *r*:

(5) $$r=\sqrt{c_{1}^{2}x^{2}+c_{2}^{2}y^{2}+c_{3}^{2}t^{2}}$$

where we have chosen *c*_1_ =1, *c*_2_ =1 and *c*_3_ is a numerical parameter with the unit “speed” (= spatial distance/time unit).

Radial basis functions have been successfully used for higher dimensions. Therefore, in this paper, we follow the more general approach of radial basis functions, use it for a problem with three dimensions (two spatial and one time dimension) and choose the inverse function 1/*r*. Other types of radial basis functions can be used, and we plan to report about such studies in a future publication.

We also need to point out that the common danger in interpolation of real-life problems is that a method cannot know about “additional” variations not accounted for in the spatial and/or temporal measurements. The common assumption is that nothing unusual happened between the measurements in space and time. If high oscillations between measurement locations really happened and have not been recorded, then this cannot be accounted for or detected in the interpolation method. We utilize some trusted large data sets to test our approach to quickly find connections to unsampled location-time pairs using assumptions reasonable for geographic data. Very reasonable for our approach is to prefer an estimation of a radius of influence in space and time instead of always using a fixed number of sampled data values, which would not take into account that points very far away with no real influence should be left out. That is why we introduced the improved IDW method in this paper. In our numerical experiments, it showed that the use of even randomly selected influence radii (*i.e*., the maximum Euclidean distance and the maximum time difference) for unsampled points leads to better interpolation results (see Section 3.4).

In summary, this article provides methodological advances, but has limited insight into the space-time patterns of air pollution that occur across the contiguous U.S. The focus of this paper is a test of the original and improved IDW methods on the large experimental PM_2.5_ dataset, by addressing computational issues with the help of parallel programming techniques and advanced data structures. For the health outcomes, there is a time delay which could be substantial, depending on the type of health problems caused by the PM_2.5_ pollution. At this point in time, we cannot provide a health problem correlation analysis for the test data we utilized. We hope that this study can be useful for future correlation studies between PM_2.5_ pollution and health outcomes.

### 2.3. Applying Parallel Computing Techniques

#### 2.3.1. Motivation of Using Parallel Computing

Initially, we implemented the IDW method using the traditional sequential algorithm where the interpolation for each location was done in sequence with one interpolation at a time. However, the sequential algorithm turned out to be too slow for very large datasets, such as the experimental PM_2.5_ data in the contiguous United States. According to Section 2.1, daily PM_2.5_ concentration values in 2009 for centroids of individual counties and census block groups need to be interpolated, which leads to 3109 × 365 = 1, 134, 785 interpolation results at the county level and 207, 630 × 365 = 75, 784, 950 interpolation results at the census block group level. Since the PM_2.5_ interpolation results are very large, it would be beneficial to apply parallel computing techniques.

Parallel computing is the ability to perform different tasks of a process simultaneously. This is achieved by using a separate thread for each task. There are several threads in a process at a time, but the processor will handle only a single thread at a time. It appears to be concurrent processing to the end user, but internally, only a single thread will be running. A processor is able to multi-task by switching between the threads. In multiprocessing, there is more than one processor, and each thread will be handled by a different processor, which leads to concurrent processing of tasks.

The IDW spatiotemporal interpolation application takes advantage of a multi-core processor. A multi-core processor has two or more processors that have been attached for enhanced performance, reduced power consumption and more efficient simultaneous processing of multiple tasks. Therefore, the performance is improved by splitting the work of the IDW spatiotemporal interpolations between multiple threads, where each thread is handled by different processors, and performing multiple interpolations simultaneously. Hence, the sequential algorithm was redesigned to use a multi-threaded parallel computing approach with a multi-core processor.

A multi-threaded program contains two or more parts that can run concurrently. Each part of such a program is called a thread, and each thread defines a separate path of execution. A thread is a single sequential flow of control within a program. Since a thread can only run once, a thread needs to be created per task. A simplistic model would create a new thread each time a request arrives and service the request in the new thread. This approach works fine for prototyping, but has significant disadvantages that would become apparent when deploying an application that worked this way. A disadvantage of the thread-per-request approach is that the overhead of creating a new thread for each request is significant; an application that created a new thread for each request would spend more time and consume more system resources creating and destroying threads than it would processing actual user requests [63]. A thread pool offers a solution to both the problem of thread life-cycle overhead and the problem of excessive resource consumption [63].

Since creating and starting new threads is computationally expensive, a cached thread pool is used in our study to improve performance for the spatiotemporal IDW interpolation method and prevent excessive resource consumption. A cached thread pool is a group of pre-instantiated and idle threads that stand ready to be given work. The cached thread pool creates new threads as needed, but will reuse previously constructed threads when they are available. A cached thread pool improves the performance of programs that execute many short-lived asynchronous tasks. When the thread pool is handed a task, it takes a previously constructed thread from the container. If no existing thread is available, a new thread is created and added to the pool. Once the thread completes the task, the thread hands itself back to the thread pool to be put into the container for re-use. Threads that have not been used for sixty seconds are terminated and removed from the cache.

In addition to the overhead of creating and destroying threads, active threads consume system resources. Creating too many threads in one Java Virtual Machine (JVM) can cause the system to run out of memory due to excessive resource consumption [63]. To prevent excessive consumption of resources, an application needs some means of limiting how many requests are being processed at any given time. In the interpolation application running on an *N*-processor machine, adding additional threads improves throughput as the number of threads approaches *N*, but adding additional threads beyond *N* will do no good. Too many threads may even degrade performance, because of the additional context switching overhead (*i.e*., the process of storing and restoring the CPU state, so that thread execution can be resumed from the same point at a later point in time). The optimum size of the thread pool depends on the number of processors available and the nature of the tasks on the work queue. On an *N*-processor system for a work queue that holds entirely compute-bound tasks, such as our interpolation work, maximum CPU utilization will be achieved with a thread pool of *N* threads. In summary, multi-threading using a thread pool enables the spatiotemporal IDW interpolation method to be more efficient by making maximum use of the CPU, because idle time can be kept to a minimum and the interpolation work is split up between threads.

#### 2.3.2. Implementation of Parallel Computing

During the interpolation, each thread takes one line of location data from the dataset to be interpolated and performs the interpolation for all the possible time instances for that line of data. For our experimental PM_2.5_ data, the datasets to be interpolated contain the centroid locations of individual counties or census block groups in the contiguous United States. Since the time domain is (2009, month, day), all of the possible time instances are the 365 days in 2009. After a thread finishes the interpolation work for the line of the location data, it retrieves the next line of location data from the interpolation queue. This is repeated, until the interpolation queue is empty. Each thread can perform the interpolation work without conflicting with other threads, because the points being interpolated are not dependent on the interpolation values for other points. Therefore, a performance advantage is gained by using threading methodology to split and share the whole interpolation work among processor cores and threads.

Our IDW interpolation application uses the Java Virtual Machine (JVM) for the multi-threading functionality. In the JVM, there is a direct mapping between a Java thread and a native operating system thread. After the JVM prepares the state for the Java thread, such as thread-local storage, allocation buffers, synchronization objects, stacks and the program counter, the native operating system thread is created. The operating system is therefore responsible for scheduling all threads and dispatching them to any available CPU. Once the native thread has initialized, it invokes the run method that contains the tasks that are executed in the Java thread. Please see Section 3.1 for the results of improvements on computational performance by using the parallel programming techniques discussed in this section.

### 2.4. k-d Tree Data Structure

#### 2.4.1. Motivation of Using k-d Tree

The most time-consuming part of the IDW interpolation method is the k-nearest neighbors search. The IDW interpolation method assumes that points that are close to one another are more alike than those that are farther apart. In order to predict a value for any unmeasured location, the IDW interpolation method uses the measured values surrounding the prediction location. Those measured values closest to the prediction location are defined as the k-nearest neighbors and have more influence on the predicted value than those farther away. Thus, IDW assumes that each measured point has a local influence that diminishes with distance.

The k-nearest neighbors are determined by calculating the Euclidean distance between the point with a known measurement and the point that is to be interpolated. The measured points that have the closest distances to the interpolation point are selected as the nearest neighbors. The number of nearest neighbors to find is a user-defined constant. The naive nearest neighbor search implementation involves the brute force computation of distances between the point to interpolate and all of the measured points in the dataset. However, as the number of samples in the dataset grows, the brute force approach quickly becomes unfeasible. Since the PM_2.5_ datasets are extremely large with more than 75 million points to interpolate at the census block group level and more than one million points to interpolate at the county level, the brute force algorithm is not a feasible implementation to use for finding the k-nearest neighbors in the IDW interpolation method.

The IDW interpolation application requires fast nearest neighbor searches in multidimensional data. An efficient solution used for this problem is the k-d tree. It has the advantage that it is easy to build and has a simple algorithm for nearest neighbor searches. A k-d tree, or k-dimensional tree, is a data structure used in computer science for organizing points in a space with *k* dimensions. k-d trees are multidimensional binary search trees [64] that are very useful for nearest neighbor searches in multidimensional data, so they have been adapted to improve the performance of the k-nearest neighbors search in our study.

#### 2.4.2. Properties of a k-d Tree

**Figure 2:**
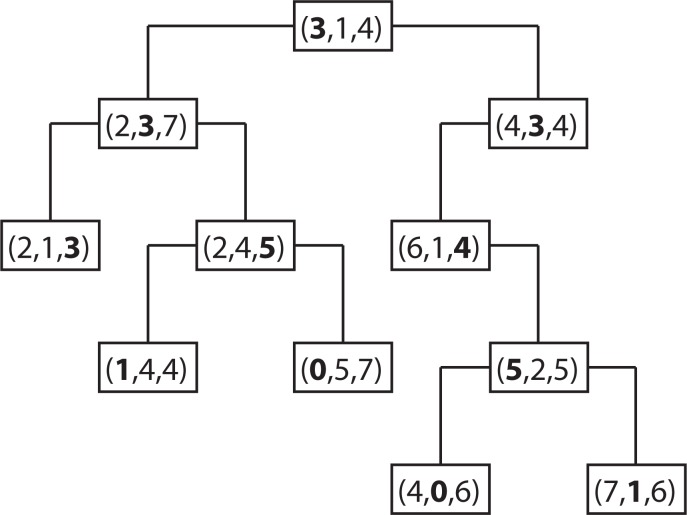
A sample k-d tree that stores points in three-dimensional space.

The k-d tree data structure is a type of binary tree that uses keys that have multiple dimensions. At a high level, a k-d tree is a generalization of a binary search tree that stores points in k-dimensional space. That is, a k-d tree can be used to store a collection of points in the Cartesian plane. While it is possible to build a k-d tree to hold data of any dimension, all of the data stored in a k-d tree must have the same dimension. Figure 2 shows an example of a k-d tree that stores 11 points in a three-dimensional space. The coordinate system in three-dimensional space needs three coordinate axes, the x-, y- and z-axis. Therefore, a point in three-dimensional space has three components (*d*_0_, *d*_1_, *d*_2_), where *d*_0_, *d*_1_ and *d*_2_ are the coordinates of the x-, y- and z-axis. In the case for the spatiotemporal IDW interpolation based on the extension approach, the z-axis is replaced by the t-axis for the spatiotemporal data.

In each level of the k-d tree shown in Figure 2, a certain component of each node has been bolded. Suppose the components are zero-indexed (for example, the x dimension is component zero, the y dimension is component one and the z or t dimension is component two). In level *n* of the tree, the (*n*%3)*rd* component of each node is shown in bold. The reason that these values are bolded is because each node acts like a binary search tree node that discriminates only along the bolded component. For example, the root of the k-d tree in Figure 2 has the value (**3**, 1, 4), with the first component, 3, in bold. The first component (x-coordinate) of every node in the k-d tree’s left sub-tree is less than or equal to three, while the first component of every node in the right sub-tree is greater than three. Similarly, consider the k-d tree’s left sub-tree. The root of this sub-tree has the value (2, **3**, 7), with the second component, 3, in bold. All of the nodes in this sub-tree’s left sub-tree have a value for the second component (y-coordinate) that is less than or equal to three, while in the right sub-tree, the second component of each node is greater than three. This trend continues throughout the tree.

#### 2.4.3. Constructing a k-d Tree

Constructing a k-d tree is fairly straightforward by partitioning point sets. Each node in a k-d tree is defined by a plane through one of the dimensions that partitions the set of points into two sets, each with half of the points of the parent node. These children are again partitioned into equal halves, using planes through a different dimension. The cutting planes along any path from the root to another node defines a unique box-shaped region of space, and each subsequent plane cuts this box into two boxes. For a set of *n* points in *k* dimensions, partitioning stops after log *n* levels, with each point in its own leaf cell. Each box-shaped region is defined by 2*k* planes, where *k* is the number of dimensions.

The k-d tree constructing algorithm selects the splitting plane in k-dimensional space by cycling through the dimensions, that is, the algorithm partitions first on *d*_0_, then *d*_1_, … , *d_k_*_−1_ before cycling back to *d*_0_. For example, in a two-dimensional k-d tree, each level in the tree alternates between the x-axis and the y-axis in order to partition the points. At the root level, all of the points to the left of the root point have smaller or equal x-values, and all of the points to the right have larger x-values. At one level lower, the tree partitions according to the y-values. Thus, all of the points in the left branch of a node have smaller or equal y-values, and all the points in the right branch have greater y-values. The next level down splits by the x-axis again, and so on.

Once the algorithm knows the axis or dimension it is working in, it goes through the list of points and finds the median point according to its value on the axis. Once the median point has been found, it is simply a matter of splitting the points up into two branches and recursively processing each branch.

**Figure 3:**
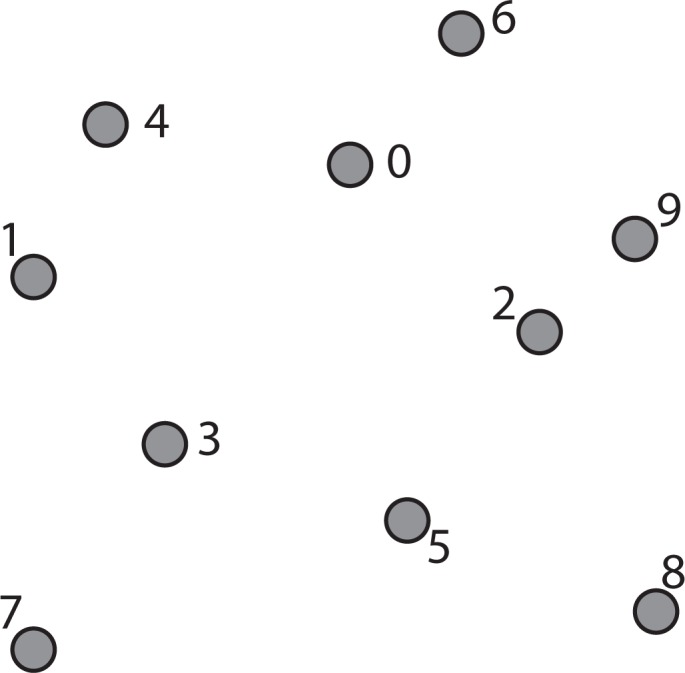
A collection of points in two-dimensional space.

**Figure 4:**
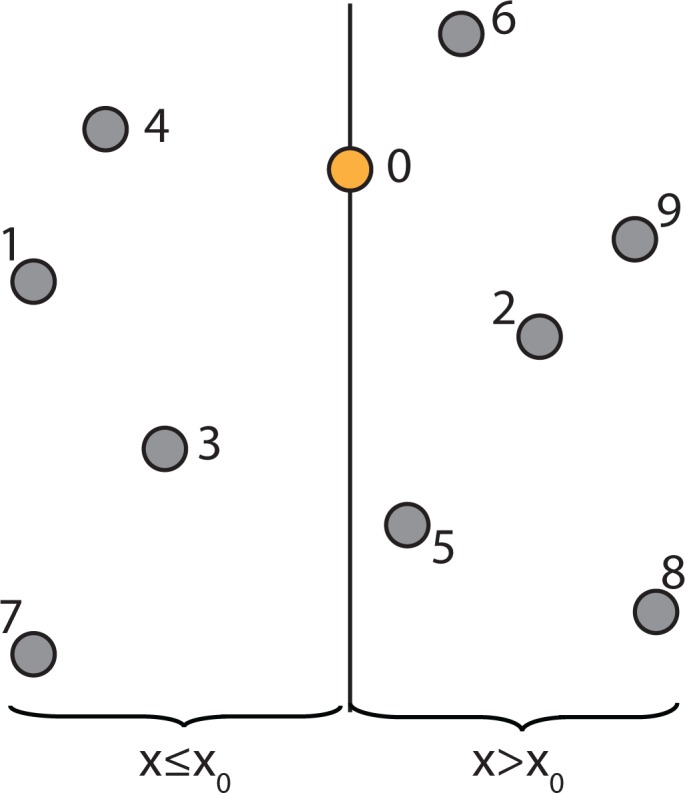
Visualization of splitting the data set from Figure 3 into two groups.

For example, a collection of points in two-dimensional space is shown in Figure 3. The constructing algorithm begins to build the k-d tree out of these data points, by choosing Node 0 (*x*_0_, *y*_0_) as the splitting node, which is the median point according to the x-axis and splitting the data set into two groups: one group with points whose x-components are less than or equal to the splitting node’s, and the other group with points whose x-components are greater than the splitting node’s. This split can be visualized in Figure 4. This is equivalent to running a splitting hyper-plane through the median Node 0 according to the x-axis.

**Figure 5:**
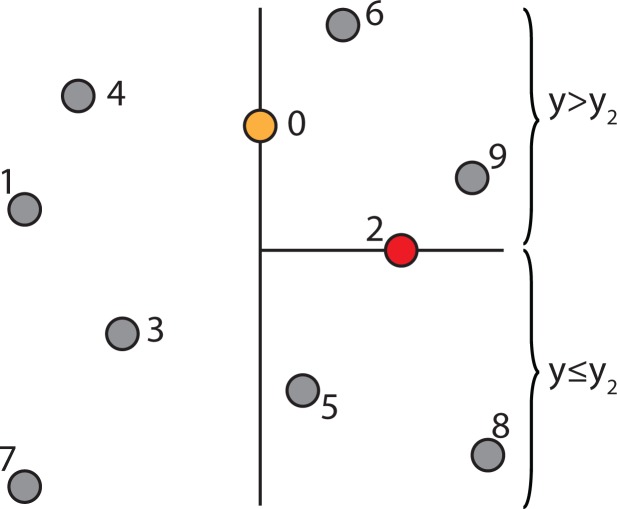
Recursively build the k-d tree in the right half-space.

**Figure 6:**
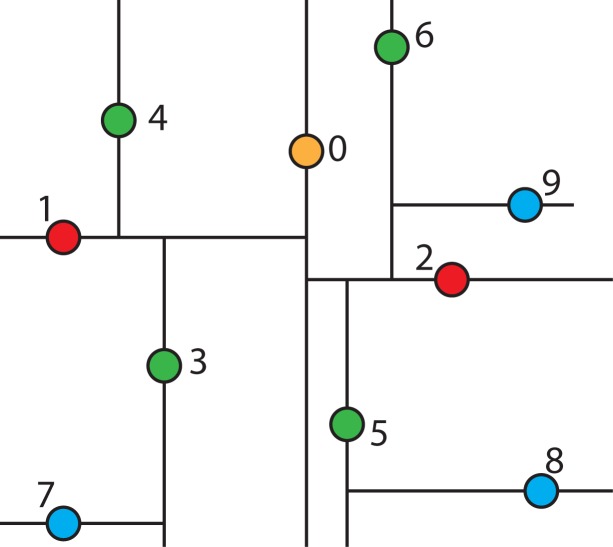
Fully constructed k-d tree.

To continue building the k-d tree, recursively build the k-d tree in the right half-space in Figure 4 (*i.e*., the points to the right of the central Node 0) by picking Node 2, which is the median point according to the y-axis and splitting the data horizontally through it, as shown in Figure 5. Continuing this partition to completion will result in the k-d tree, as shown in Figure 6.

**Figure 7:**
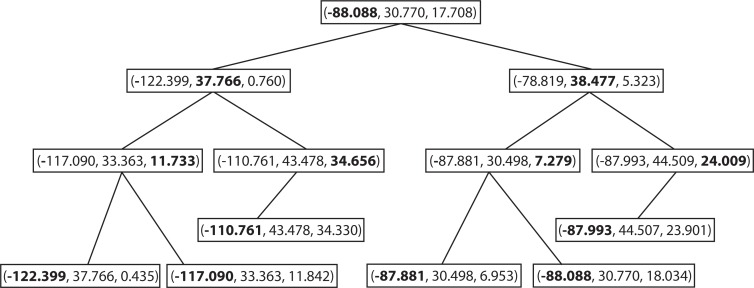
A sample k-d tree constructed from some selected PM_2.5_ data points.

For the spatiotemporal IDW interpolation for the PM_2.5_ data, a three-dimensional k-d tree has been constructed to find the k-nearest neighbors. Figure 7 illustrates a sample k-d tree constructed from some selected PM_2.5_ data points.

#### 2.4.4. Searching a k-d Tree

**Figure 8:**
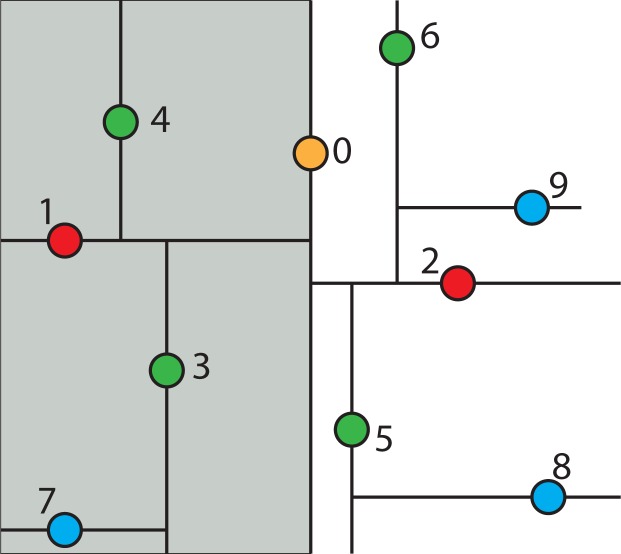
The left half-space of k-tree is ignored when searching Node 8.

To give a better sense for the geometric intuition behind the k-d tree, the following example will trace through what happens when looking up whether a given point is in the k-d tree. Suppose the query point is Node 8 in Figure 6. Then, begin at the root of the k-d tree and consider whether Node 8’s x-coordinate is less than or greater than the root Node 0’s x-coordinate. This is equivalent to asking in which half-space Node 8 is. The node happens to be in the right half-space, and so, all of the nodes in the left half-space can be ignored. Therefore, the right half-space is recursively explored. This is shown graphically in Figure 8, where the grayed-out region corresponds to parts of the plane that will never be looked in.

**Figure 9:**
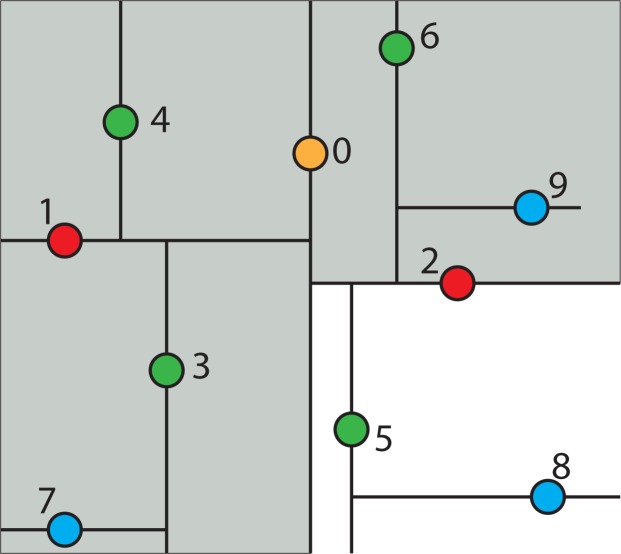
The top half-space in the right sub-tree is ignored when searching Node 8.

Now, check whether Node 8 is above or below Node 2, which is the root of the subtree in the half-space to the right of Node 0. Node 8 is below it, so discard the top half-space and look in the bottom, as shown in Figure 9.

**Figure 10:**
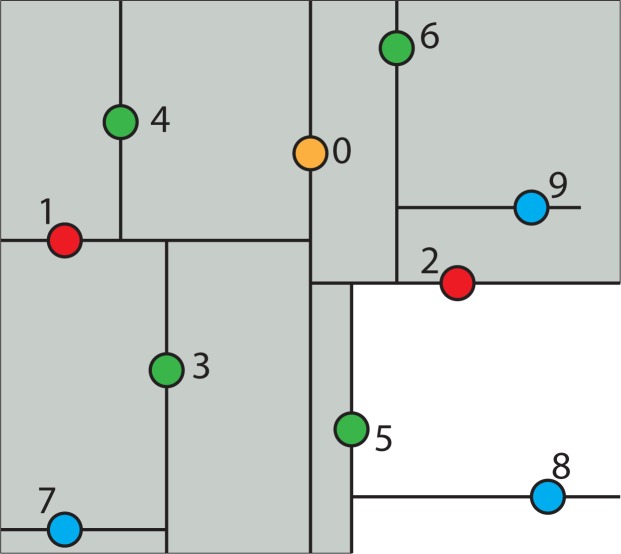
The half-space to the left of Node 5 is ignored when searching Node 8.

Next, check whether the query point, Node 8, is to the left or the right of Node 5. Node 5 is the root of this region of space. Since, the query point is to the right, discard the sliver of a half-space to the left of Node 5 and continue on as shown in Figure 10.

At this point, the algorithm has reached the query Point 8 that is being looked for, and the k-d tree searching algorithm terminates.

#### 2.4.5. Nearest Neighbor Search Algorithm using k-d Tree to Find One Nearest Neighbor

The nearest neighbor search algorithm is used to find the point in the tree that is nearest to the given point. This search can be done efficiently by using the k-d tree properties to quickly eliminate large portions of the search space. The k-d tree data structure hierarchically decomposes space into a small number of cells, each containing a few representatives from an input set of points. This provides a fast way to find the point closest to the query point *q*.

**Figure 11:**
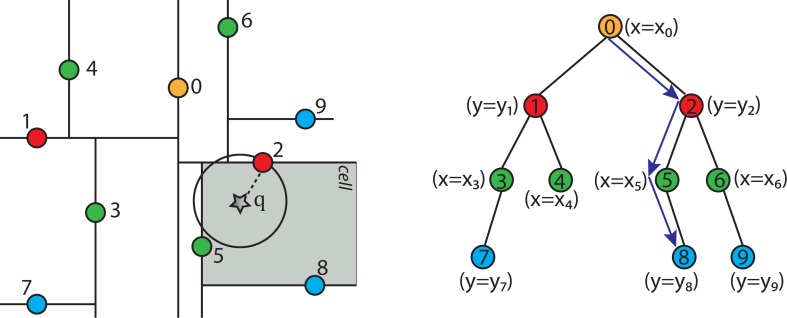
A fully constructed k-d tree and its tree structure.

Figure 11 shows the fully constructed k-d tree partition as in Figure 6 with an additional query point *q* indicated by a star and its tree structure. Each line segment in the k-d tree space partition represents a branch in the tree, which effectively divides each sub-tree into two halves. The geometric function of the splitting hyper-line (in a 3D case, it would be a splitting hyper-plane) for each partition is given in the parenthesis next to each node in the tree structure of the figure.

To find the nearest neighbor for a given point *q* using the k-d tree shown in Figure 11, the nearest neighbor search algorithm needs to search through the tree in an appropriate manner. The key to the k-d tree traversal in a nearest neighbor search is in deciding on the right branch to explore. Since each branch in a k-d tree represents a space partition, the idea is to explore the partition that is closer to the query point first. The partition that is closer to the query point will likely contain the nearest neighbor.

The nearest neighbor search algorithm works by starting at the root node and walking down the k-d tree recursively as if it were searching the tree for the query point. The path of walking down the tree to search the query point *q* is illustrated by arrowed lines in Figure 11. Once the algorithm reaches a leaf node in the k-d tree (*i.e*., Node 8 in Figure 11), it saves that node as the current best nearest neighbor. As the algorithm starts unwinding the recursion and walking back up the tree, it checks whether each node on the path is better than the best estimate it has seen so far. If the current node is determined to be better than the previous node, the algorithm updates the best estimate to the current node.

During each step of walking up the tree, the algorithm must decide which, if any, neighboring cells/nodes need to be checked, as well. This is done by forming a hyper-sphere centered at the query point with the radius of the hyper-sphere being the calculated distance between the query point and the current best nearest neighbor. The algorithm then checks whether the candidate hyper-sphere based on the current best estimate could cross any of the splitting hyper-planes that form the cell. If the candidate hyper-sphere does not cross the splitting hyper-plane, then the algorithm eliminates all points on the other side of the splitting hyper-plane from consideration and walks back up to the next node on the path in the k-d tree. Otherwise, it means there is a possibility that there is a closer point in cells on the other side of the splitting hyper-plane. Therefore, the algorithm must look on the other side of the hyper-plane of the k-d tree to determine if there are any closer points, following the same recursive process as the entire search.

If there is a point in the data set that is closer to the query point than the current best guess, then it must lie in the circle centered at the query point that passes through the current best guess (for example, Node 2), as shown in Figure 11. Although in this example, this region is a circle, in three dimensions, it would be a sphere, as is the case for the PM_2.5_ data. The sphere is called the candidate hypersphere.

**Figure 12:**
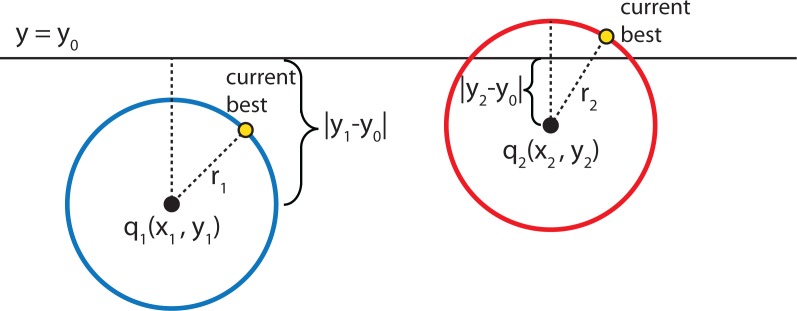
The left hyper-circle does not cross the hyper-line, but the right hyper-circle does cross the hyper-line.

Given a circle and a line (or a hyper-sphere and a hyper-plane), how does the algorithm determine whether or not the circle intersects the line? To determine this mathematically, consider the following arbitrary hyper-line and two hyper-circles, one of which crosses the hyper-line and one of which does not, as shown in Figure 12.

Figure 12 shows that the distance |*y*_1_ − *y*_0_| from the center of the left hyper-circle to the hyper-line is greater than the radius of the left hyper-circle, and therefore, the hyper-circle does not cross the hyper-line. However, the distance from the center of the right hyper-circle to the hyper-line is less than the radius of the right hyper-circle, and therefore, some part of that hyper-circle does cross the hyper-line. Hence, for the nearest neighbor searching algorithm to work properly, it is essential to check the other side of the right hyper-line for potentially closer nearest neighbors.

In general, to check if a hyper-plane intersects with a hyper-sphere of radius *r* centered at the query point *q*(*q*_0_, *q*_1_, *q*_2_,...,*q_k_*_−1_) in a k-dimensional space, the algorithm compares the radius of the hyper-sphere to the distance from *q* to the partition plane. If node *a*(*a*_0_, *a*_1_,*a*_2_,...,*a_k_*_−1_) partitions points based on their *i*-th dimension, then the hyper-sphere crosses the node’s splitting plane only if:

(6) $$\left|q_{i}-a_{i}\right|<r$$

Therefore, the following steps are followed to find the nearest neighbor for a given query point:

- Given a current best estimate of the node that may be the nearest neighbor, a candidate hyper-sphere can be constructed that is centered at the query point *q*(*q*_0_, *q*_1_, *q*_2_,...,*q_k_*_−1_) and running through the current best node point. The nearest neighbor to the query point must lie inside the hyper-sphere.
- If the hyper-sphere is fully to one side of a splitting hyper-plane, then all points on the other side of the splitting hyper-plane cannot be contained in the sphere and, thus, cannot be the nearest neighbor.
- To determine whether the candidate hyper-sphere crosses the splitting hyper-plane that compares coordinate at dimension *i*, check whether |*q_i_* − *a_i_*| < *r*.

#### 2.4.6. Adapted Neighbor Search Algorithm Using a k-d Tree to Find Multiple Nearest Neighbors

The original nearest neighbor searching algorithm using the k-d tree data structure can be found in [65]. It find one nearest neighbors. The original algorithm was adapted to make it more efficient for searching the PM_2.5_ data to find multiple (*k*) nearest neighbors of a given data point *q* instead of just finding one nearest neighbor. The modified algorithm uses a data structure called a bounded priority queue that stores the list of k nearest neighbors with their distances to the query point *q*. The bounded priority queue has a fixed upper bound on the number of elements (or points) that can be stored, which is the number of nearest neighbors *k*. Whenever a new element is added to the queue, if the queue is at capacity, the element with the highest priority value (*i.e*., the longest distance) is ejected from the queue.

**Figure 13:**
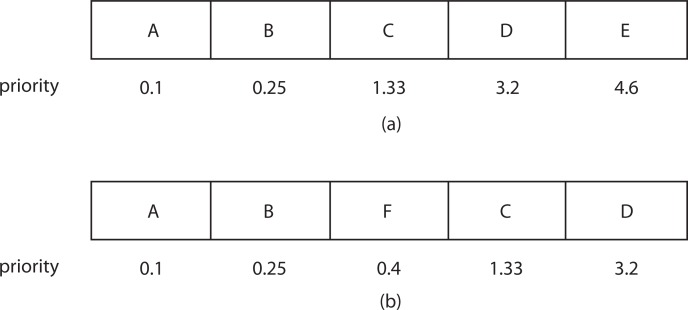
Bounded priority queue for k-nearest neighbors.

Figure 13a shows a nearest neighbor priority queue that has a maximum size of five and holds five elements, A–E. Suppose that the next nearest neighbor to be inserted into the priority queue is the Element F with a priority or distance of 0.4. Since the priority queue has a maximum size of five, the Element F is inserted into the priority queue, but the Element E with the longest distance is deleted from the priority queue. Figure 13b shows the resulting priority queue after Element F is inserted. In another case, suppose that the next nearest neighbor to be inserted into the priority queue is the Element G with a distance of 4.0. Since the distance value for G is greater than the maximum priority element in the priority queue, G will not be inserted into the queue.

Using the bounded priority queue to store nearest neighbors, the pseudocode for the adapted k-nearest neighbor search using the k-d tree is given in Algorithms 1–3. This method *getNearestNeighbors* in Algorithm 1 calls the *searchNode* method in Algorithms 2 and 3 initially on the leaf node. After the *searchNode* method finishes with the leaf node, it returns to the *getNearestNeighbors* method, which moves to the leaf’s parent and calls the *searchNode* method on the parent, and so on.

**Table:** 

**Algorithm 1: getNearestNeighbors(k, value)** k-nearest neighbor search in a k-d tree
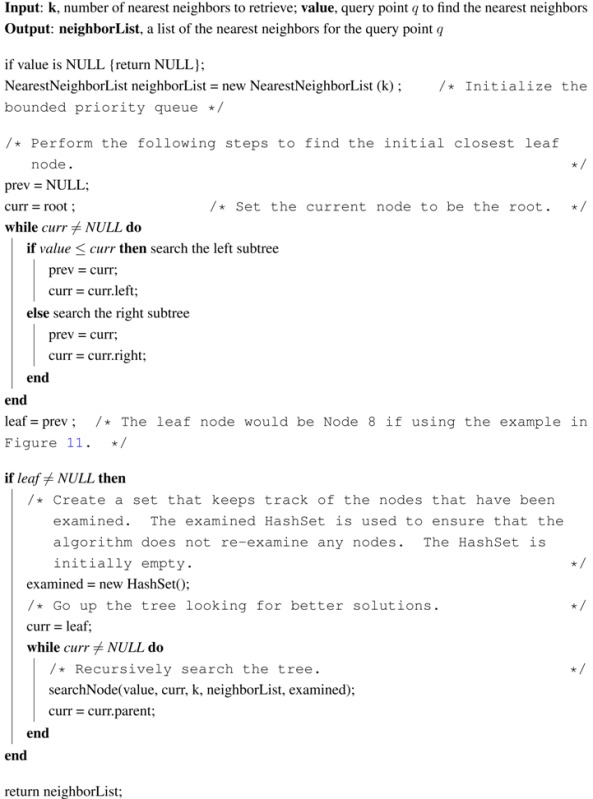
**Algorithm 2: searchNode(value, curr, k, neighborList, examined), Part I** moving up the k-d tree to look for better nearest neighbors
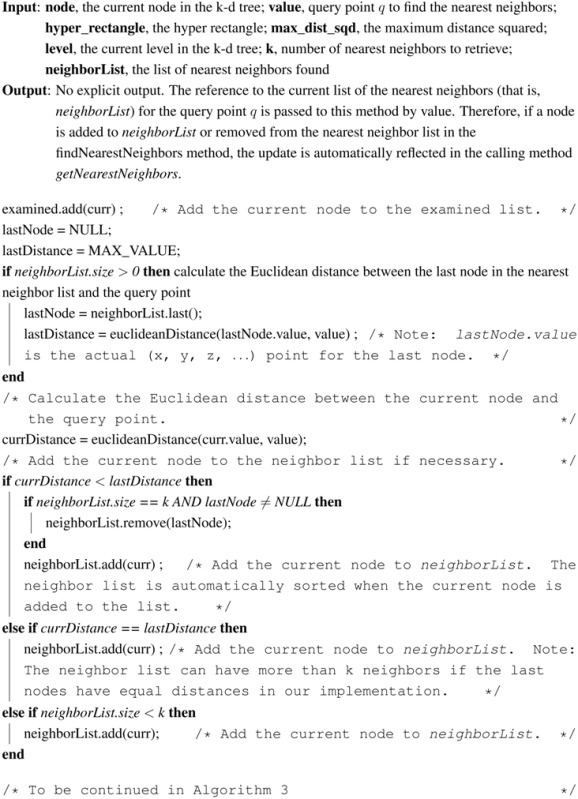
**Algorithm 3: searchNode(value, curr, k, neighborList, examined), Part II**
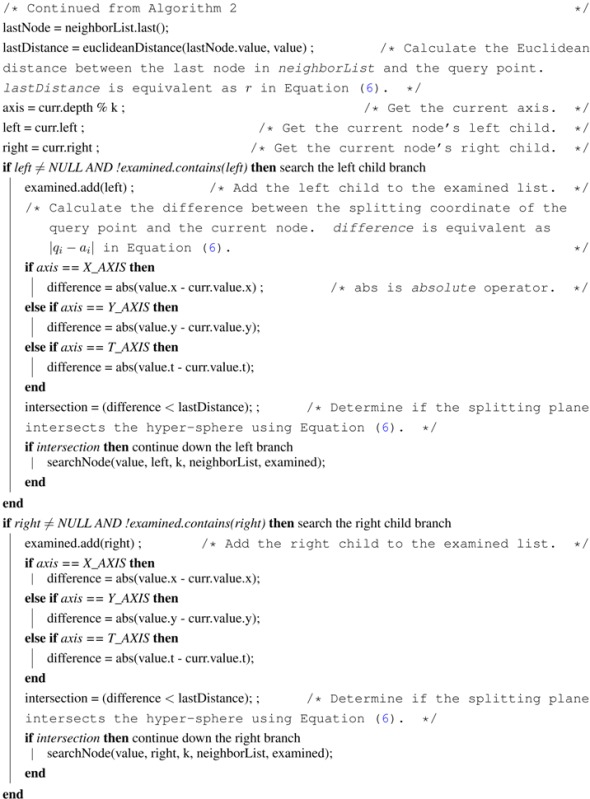

The main idea of the *searchNode* method in Algorithms 2 and 3 is to move up the k-d tree and look for better solutions for nearest neighbors than the current best node, which is initially the closest leaf node (the variable *leaf* in Algorithm 1). If the neighbor list already has k nearest neighbors, then check the last node in the list to ensure that the distance from the query point is less than the current node distance. If the last node has a distance greater than the current node, then remove the last node from the list and add the current node. Otherwise, if the neighbor list is not full, then add the current node to the list.

Next, in the *searchNode* method, the algorithm checks whether there could be any points on the other side of the splitting plane that are closer to the query point than the last node in the neighborList. This is done by intersecting the splitting hyper-plane with a hyper-sphere around the query point that has a radius equal to the distance from the query point to the last node in the list of nearest neighbors. Since the hyper-planes are all axis-aligned, this is implemented as a simple comparison to see whether the difference between the splitting coordinate of the query point and current node, which is |*q_i_* − *a_i_*| in Equation (6), is less than the Euclidean distance from the query point to the last node in the list of nearest neighbors, which is *r* in Equation (6). If the hyper-sphere crosses the plane, there could be nearer points on the other side of the plane, so the algorithm must move down the other branch of the tree from the current node looking for closer points, following the same recursive process as the entire search.

In summary, there are two changes to this algorithm that differentiate it from the conventional one-nearest neighbor searching algorithm. The first change is when determining whether to look on the opposite side of the splitting hyper-plane, the algorithm uses the distance from the query point to the point with the longest distance in the nearest neighbor priority queue as the radius of the candidate hyper-sphere. This is required when finding the k-nearest neighbors, because the candidate hyper-sphere for the k-nearest points needs to encompass all k of those neighbors and not just the closest neighbor. The other change is that when the algorithm considers whether to look on the opposite side of the splitting hyper-plane, the algorithm takes into account whether the bounded priority queue for storing nearest neighbors contains at least k points. This is extremely important, because if parts of the tree are pruned off before there is at least k points, one of the closest points may be accidentally thrown out.

The k-nearest neighbor search algorithm can run in O(log n) time on a balanced k-d tree with *n* data points provided that the points are randomly distributed [66]. In the worst case, the entire tree may need to be searched. However, in low-dimensional spaces, such as the Cartesian plane or three-dimensional space, the entire k-d tree is rarely searched.

Section 3.2 gives the improvement results on computational performance after using the adapted k-d tree to search for k nearest neighbors.

### 2.5. Cross-Validation

Cross-validation [67] is a model evaluation method that is better than residuals [68]. In statistics, a residual refers to the amount of variability in a dependent variable that is left over after accounting for the variability explained by the predictors in the analysis. For example, when predictors or independent variables are included in a regression, a prediction is made that they are associated with a dependent variable. A residual is a numeric value for how much the prediction is wrong. The lower the residual, the more accurate the predictions in a regression are, indicating that the independent variables are predictive of the dependent variables. The problem with residual evaluations is that they do not give an indication of how well the learner will do when it is asked to make new predictions for data it has not already seen. One way to overcome this problem is to not use the entire dataset when training a learner. Some of the data is removed before training begins. Then, when training is done, the data that was removed can be used to test the performance of the learned model on new data. This is the basic idea for a whole class of model evaluation methods called cross-validation.

In our study, K-fold cross-validation (KFOLDCV) using 10-folds and leave-one-out cross-validation (LOOCV) were compared for determining an optimal IDW method for interpolating the PM_2.5_ concentration values based on the number of nearest neighbors *N* and the exponent *p*. The effect of changing *p* and *N* was investigated by previewing the output of the IDW interpolation methods and calculating error statistics using KFOLDCV and LOOCV.

#### 2.5.1. K-Fold Cross-Validation Method

Cross-validation splits the data, once or several times, for estimating the performance of each interpolation method. Part of the data is used as the training sample, and the remaining part is used as the validation sample. The validation sample is used for estimating the performance of the interpolation method. The interpolation method with the smallest error is selected as the best method.

In k-fold cross-validation, the dataset is divided into *k* subsets. Each time, one of the *k* subsets is used as the test set (validation set), and the other *k* − 1 subsets are put together to form a training set. The average error across all *k* trials is computed. The advantage of this method is that it matters less how the data gets divided. Every data point gets to be in a test set exactly once and gets to be in a training set *k* − 1 times. The variance of the resulting estimate is reduced as *k* is increased. The disadvantage of this method is that the training algorithm has to be rerun from scratch *k* times, which means it takes *k* times as much computation to make an evaluation.

#### 2.5.2. Leave-One-Out Cross-Validation Method

Leave-one-out cross validation is the degenerate case of k-Fold cross validation, where k is chosen as the total number of samples. Leave-one-out cross-validation performs N experiments for a dataset with N samples. For each experiment, N-1 samples are used for training, and the remaining sample is used for testing. That means that for N separate times, the function approximator is trained on all of the data except for one point and an interpolation is made for that point. Leave-one-out cross-validation has unbiased performance estimation, but has a very large variance that can cause unreliable estimates [67].

#### 2.5.3. Error Statistics

In our study, 10-fold cross-validation and leave-one-out cross-validation were implemented to evaluate the IDW spatiotemporal interpolation method given in Equation (1) with the time values as shown in the third column in Table 1. The daily fine particulate matter PM_2.5_ concentration data for the contiguous U.S. over the year of 2009 was used in the cross-validations to determine an optimal method for the number of nearest neighbors *N* and the exponent *p* in the IDW interpolation. Cross-validations were performed on the following 45 IDW methods with each method having a different number of nearest neighbors *N* and a different exponent *p*, with *N* ∈{3, 4, 5, 6, 7} and *p* ∈{1, 1.5, 2, 2.5, 3, 3.5, 4, 4.5, 5}.

Each of the 45 IDW methods were evaluated by calculating the error measurement for MARE (mean absolute relative error) and the error measurement for RMSPE (root mean square percentage error). MARE and RMSPE are defined as:

(7) $$MARE=\frac{\sum_{i=1}^{N}\frac{\left|I_{i}-O_{i}\right|}{O_{i}}}{N}$$

(8) $$\mathrm{RMSPE}=\sqrt{\frac{{\sum_{i=1}^{N}\left(\frac{\left(O_{i}-I_{i}\right)}{O_{i}}\right)}^{2}}{N}}*100$$

where *N* is the number of observations, *I_i_*s are the interpolated values, and *O_i_*s are the original values from the daily fine particulate matter PM_2.5_ concentration dataset described in Section 2.1. The optimal number of nearest neighbors and exponent combination for the PM_2.5_ data can be determined by minimizing the MARE error measurement or the RMSPE error measurement.

## 3. Results

### 3.1. Computational Performance Improvement by Using Parallel Computing

After applying the parallel programming techniques described in Section 2.3, the execution time of the IDW spatiotemporal interpolation method is reduced, and good performance speedup is achieved.

**Figure 14:**
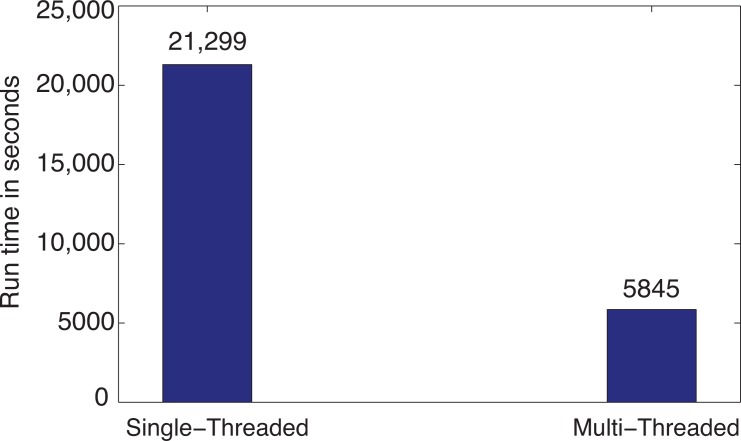
Effect of parallel computing on inverse distance weighting (IDW) run time using the PM_2.5_ data at the county level.

A test was run on a system that was equipped with an Intel Core i7-3630 QM CPU running at 2.40 GHz with 6 GB of RAM and eight available processors for servicing thread requests. Using the location dataset to be interpolated at the county level, for 1,134,785 (3109 × 365) PM_2.5_ interpolation results, the process took 21,299 seconds (355 min) using a single thread and the sequential algorithm, as illustrated by the first bar in Figure 14. Because of this unacceptable performance, the multi-threaded approach described in Section 2.3 was employed. This improved the performance by **3**.**64** times. As seen in the second bar in Figure 14, run time was reduced to 5,845 seconds on the same computer using the same dataset.

A test was also run on the same system using the dataset to be interpolated at the census block group level. For 75,784,950 (207, 630 × 365) PM_2.5_ interpolation results, using the single thread approach and the sequential algorithm, the run time was too long to be measured. Unfortunately, using the multi-threaded approach, the run time was also too long for the interpolation at the census block group level. This unacceptable performance is mainly caused by the brute force search for k-nearest neighbors in large datasets. The brute force nearest neighbor search forces the computation of spatiotemporal Euclidean distances in Equation (2) between the point to interpolate and all of the measured points and the storage of all these distance values into the computer’s memory.

Therefore, to improve the performance of the k-nearest neighbor search, the k-d tree data structure was adapted. The resulted further computational performance improvement is described in the next section, Section 3.2.

### 3.2. Computational Performance Improvement of by Using k-d Tree

By using the adapted k-d tree data structure to search multiple nearest neighbors described in Section 2.4.6, the performance of the IDW interpolations is further improved dramatically.

**Figure 15:**
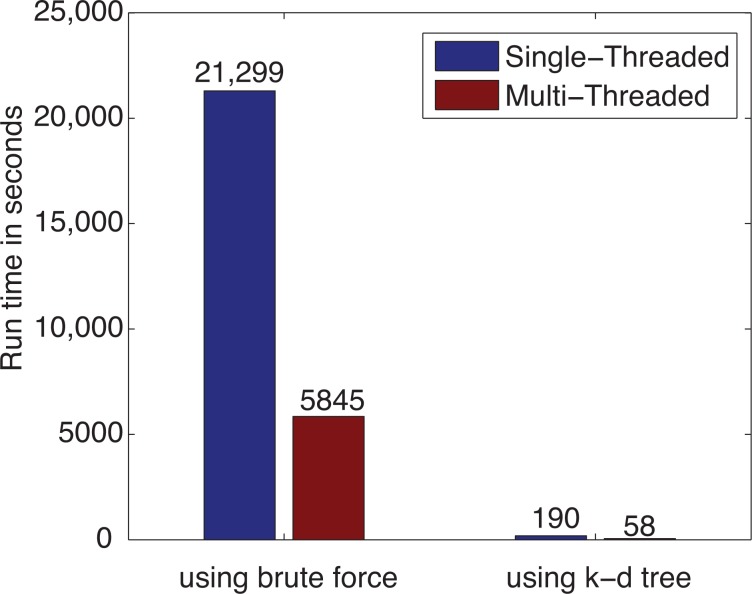
The effect of k-d tree with paralleling programming on IDW run time using the PM_2.5_ data at the county level.

The test was run on the same system as in Section 3.1 that was equipped with an Intel Core i7-3630 QM CPU running at 2.40 GHz with 6 GB of RAM. Using the PM_2.5_ location dataset to be interpolated at the county level, for 1,134,785 (3109×365) interpolation results, the interpolation using the k-d tree took only 190 s with the single-threaded approach and 58 s using the multi-threaded approach, as illustrated in Figure 15. Compared with the brute force approach to find the nearest neighbors, using the adapted k-d tree reduced the run time by **112**-times for the single-threaded approach and by **101**-times for the multi-threaded approach. It is also worthy mentioning that when k-d tree was used, the multi-thread parallel programming approach improved the computational performance from 190 s to 58 s, which is **3**.**28** times faster.

**Figure 16:**
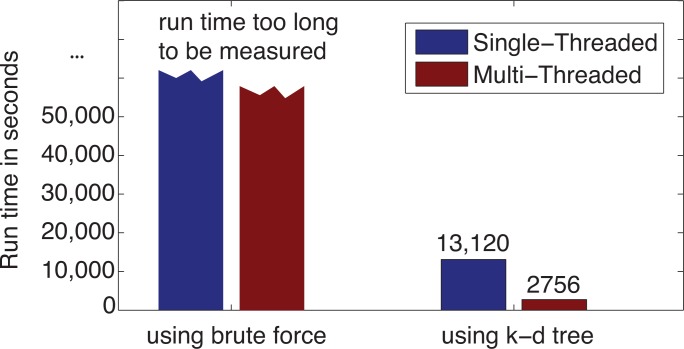
The effect of k-d tree with paralleling programming on IDW run time using the PM_2.5_ data at the census block group level.

A test was also run on the same system using the PM_2.5_ data to be interpolated at the census block group level. As mentioned in Section 3.1, for 75,784,950 (207, 630 × 365) interpolation results, the run time was too long to be measured using the brute force approach to find the nearest neighbors, even with the help of parallel computing. However, using the adapted k-d tree and single-threaded approach, the interpolation took 13,120 s (218.67 min). Using the adapted k-d tree combined with parallel programming techniques, we reduced the run time by **4.76**-times and achieved a great performance of 2756 s (46 min) for computing 75.78 million PM_2.5_ interpolation results at the census block group level, as shown in Figure 16.

### 3.3. Leave-One-Out Cross-Validation Results for the Original IDW-Based Method

**Figure 17:**
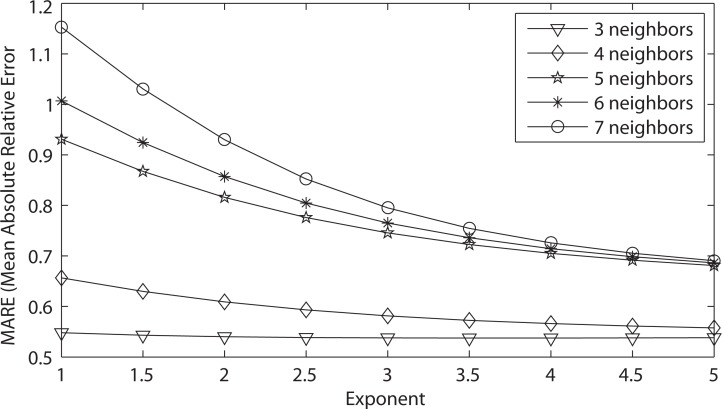
MARE LOOCV results for various 45 IDW methods using the original IDW-based interpolation.

The original IDW interpolation method uses the same number of k-nearest neighbors for all interpolation results, as shown in Equation (1). The number of nearest neighbors to find are specified by the user. Therefore, the list of nearest neighbors found will always be *k* regardless of how far away the neighbor is from the query point. Figure 17 illustrates the MARE (mean absolute relative error) results for the 45 IDW methods that were evaluated in the leave-one-out cross-validation for the original IDW-based interpolation in Section 2.2.1, using the PM_2.5_ data.

It can be concluded from Figure 17 that the best IDW methods for the PM_2.5_ data are the ones with three nearest neighbors, because they minimize the mean absolute relative error (MARE) from the cross-validation error statistics. Table 2 gives the detailed MARE values for nine IDW methods with three nearest neighbors, but different exponents. It is shown from Table 2 that of these nine IDW methods, the three nearest neighbors with an exponent of 3.5 have the lowest MARE of 0.53739.

**Table 2:** MARE LOOCV results for nine IDW methods with three nearest neighbors using the original IDW-based interpolation.

**Neighbors**	**Exponent**	**MARE**
3	1.0	0.54797
3	1.5	0.54301
3	2.0	0.54012
3	2.5	0.53849
3	3.0	0.53768
**3**	**3.5**	**0.53739**
3	4.0	0.53742
3	4.5	0.53768
3	5.0	0.53807

### 3.4. Cross-Validation Results for the Improved IDW-Based Method

The improved IDW interpolation method in Section 2.2.2 uses two parameters for the maximum Euclidean distance and the maximum time difference that the nearest neighbors can have from the query point that is to be interpolated. Therefore, the number of k-nearest neighbors is the maximum that can be found, but less may be found if the neighbor does not satisfy the maximum Euclidean distance and maximum time difference criteria.

#### 3.4.1. Leave-One-Out Cross-Validation Results

**Figure 18:**
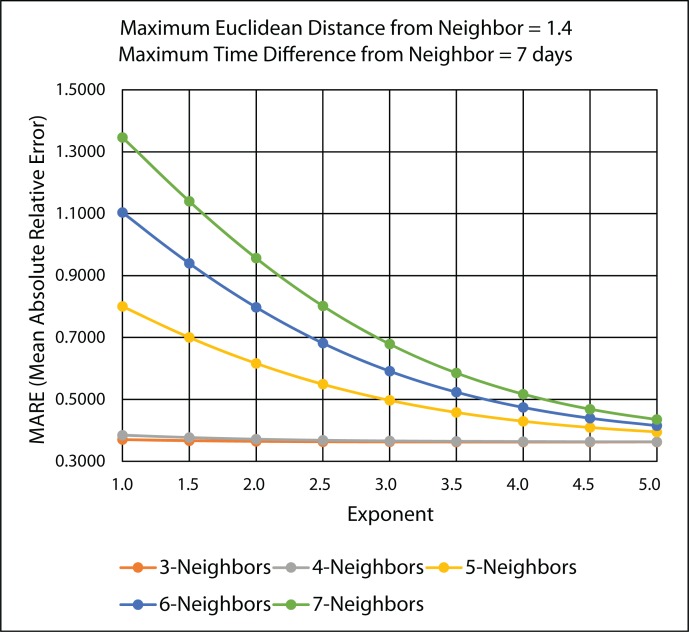
MARE LOOCV results for the various 45 IDW methods using the improved IDW interpolation.

**Figure 19:**
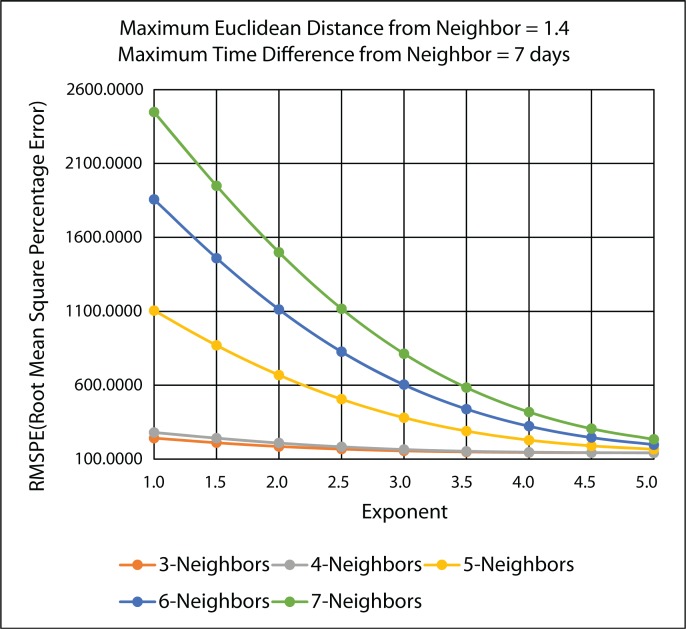
RMSPE (root mean square percentage error) LOOCV results for the various 45 IDW methods using the improved IDW interpolation.

Figure 18 illustrates the MARE (mean absolute relative error) results and Figure 19 illustrates the RMSPE (root mean square percentage error) results in the improved IDW interpolation for the 45 IDW methods that were evaluated in the leave-one-out cross validation, using the PM_2.5_ data. The maximum Euclidean distance that was used for the nearest neighbor criteria is 1.4, and the maximum time difference for the nearest neighbor criteria is seven days. These two parameter values were chosen arbitrarily to show the improvements that are achieved in the improved IDW-based interpolation method. Better results could be further achieved by choosing better maximum Euclidean distance and time difference values. Any of the k-nearest neighbors that are found that do not satisfy the maximum Euclidean distance and the maximum time difference are rejected and are not used as a neighbor in the improved IDW interpolation. Adding these two parameters to the improved IDW-based interpolation method significantly improves the MARE results shown in Figure 18 when compared with the MARE results shown in Figure 17 from the original IDW-based interpolation method.

It can be concluded from Figure 18 and Figure 19 that the best IDW methods for the PM_2.5_ data are the ones with a maximum three and four nearest neighbors, because they minimize MARE and RMSPE from the cross-validation error statistics. Table 3 gives the detailed MARE and RMSPE values for nine IDW methods with a maximum of three and four nearest neighbors, but different exponents.

**Table 3:** MARE and RMSPE LOOCV results for nine IDW methods with a maximum of three and four nearest neighbors using the improved IDW interpolation.

**Exponent**	**MARE: n = 3**	**MARE:n=4**	**RMSPE:n=3**	**RMSPE:n=4**
1.0	0.37027	0.38444	242.1428	279.8240
1.5	0.36641	0.37664	210.7435	241.7803
2.0	0.36423	0.37154	185.4772	208.6938
2.5	0.36302	0.36816	167.2276	182.7473
3.0	0.36240	0.36595	155.4234	164.5911
3.5	0.36214	0.36454	148.5401	153.2638
4.0	0.36209	0.36367	144.8794	146.9017
4.5	0.36219	0.36317	143.0899	143.6380
**5.0**	**0.36237**	**0.36294**	**142.2915**	**142.0947**

It is shown from Table 3 that of these nine IDW methods, the IDW methods with the maximum of three or four nearest neighbors and with an exponent of 5.0 (*n* =3, 4 and *p* =5.0) have the lowest MARE and RMSPE values, according to the leave-one-out cross-validation.

#### 3.4.2. Ten-Fold Cross-Validation Results

**Figure 20:**
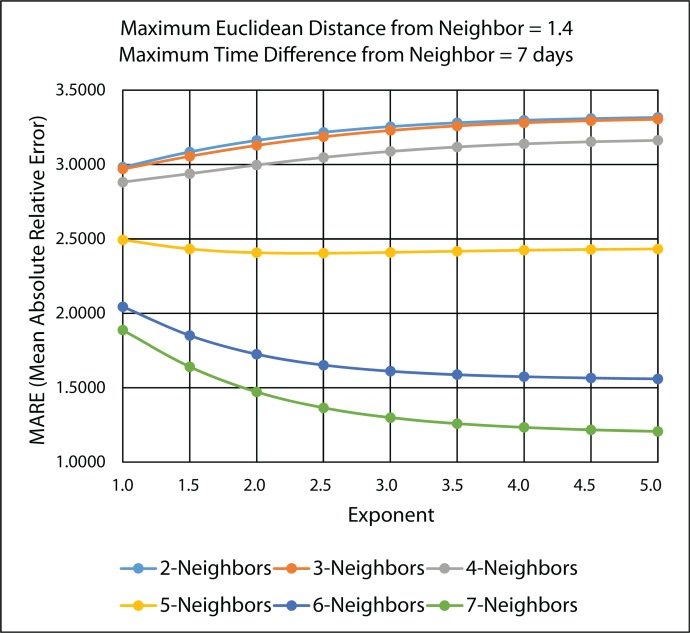
MARE 10-fold-cross validation results for the various 45 IDW methods using the improved IDW interpolation.

**Figure 21:**
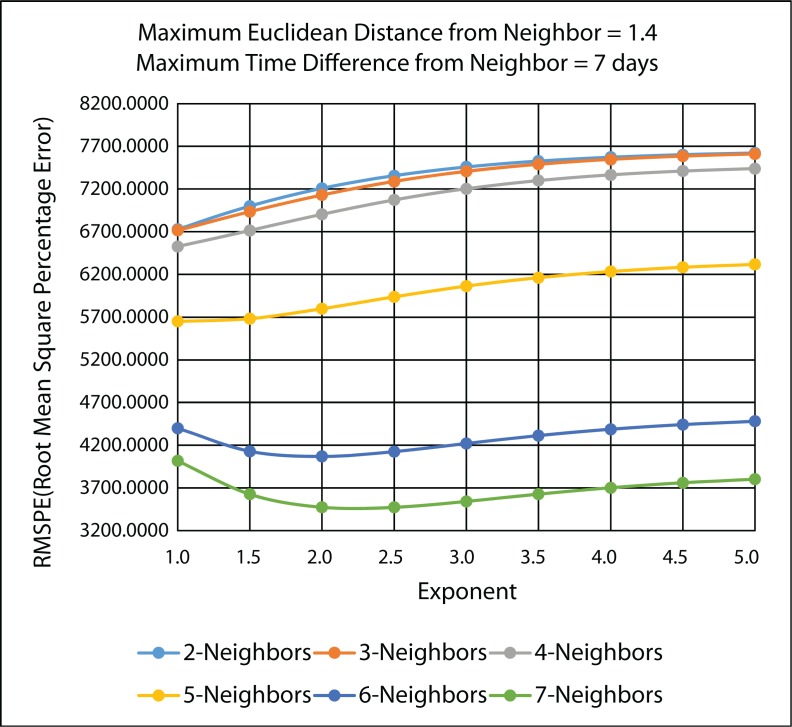
RMSPE 10-fold cross-validation results for the various 45 IDW methods using the improved IDW interpolation.

Figure 20 illustrates the MARE results and Figure 21 illustrates the RMSPE results in the improved IDW interpolation for the 45 IDW methods that were evaluated in the 10-fold cross-validation, using the PM_2.5_ data. As in the leave-one-out cross-validation, the maximum Euclidean distance that was used for the nearest neighbor criteria is 1.4, and the maximum time difference for the nearest neighbor criteria is seven days. Therefore, any of the k-nearest neighbors that are found that do not satisfy the maximum Euclidean distance and the maximum time difference are rejected and are not used as a neighbor in the IDW interpolation.

It can be concluded from Figure 20 and Figure 21 that the best IDW methods for the PM_2.5_ data are the ones with a maximum seven nearest neighbors, because they minimize the MARE and the RMSPE from the 10-fold cross-validation error statistics. Table 4 gives the detailed MARE and RMSPE values for nine IDW methods with a maximum of seven neighbors, but different exponents.

**Table 4:** MARE and RMSPE results for nine IDW methods with a maximum of seven nearest neighbors using the improved IDW interpolation.

**Exponent**	**MARE: n = 7**	**RMSPE: n = 7**
1.0	1.88664	4016.4020
1.5	1.64071	3626.1590
2.0	1.47143	3472.6450
2.5	1.36437	3472.7490
3.0	1.29870	3542.2100
3.5	1.25827	3627.0180
4.0	1.23285	3702.0150
4.5	1.21648	3759.8730
**5.0**	**1.20577**	**3801.4410**

It is shown from Table 4 that of these nine IDW methods, the maximum of seven nearest neighbors with an exponent of 5.0 has the lowest MARE and RMSPE values. Therefore, these settings (*n* =7 and *p* =5.0) should be used as the improved IDW method to interpolate the PM_2.5_ data according to the 10-fold cross-validation.

#### 3.4.3. Comparison of Leave-One-Out and 10-Fold Cross-Validation Results

Based on Table 3, using the improved IDW-based method significantly improves the performance, compared with using the original IDW-based method. The MARE is approximately 0.54 when using the original IDW-based method with the best setting of three nearest neighbors with an exponent of 3.5. However, the MARE is significantly reduced to approximately 0.36, when using the improved IDW-based method with the best setting of a maximum of three or four nearest neighbors and with an exponent of 5.0. Using the fixed number of nearest-neighbors approach, as in the original IDW-based interpolation, will always find neighbors, but they may be so far away from the interpolation points that the IDW interpolation may be giving misleading information. Therefore, it is better to limit the number of measured values by specifying a search neighborhood as in the improved IDW-based interpolation. The search neighborhood restricts how far and where to look for the measured values to be used in the interpolation. The improved IDW-based interpolation is able to achieve better performance, because neighbors that are far away are excluded from the interpolation, even if they are found to be nearest neighbors.

Based on Table 3 and Table 4, the leave-one-out and 10-fold cross-validations indicate different best settings for the improved IDW-based method. The improved IDW methods with the maximum of three or four nearest neighbors and an exponent of 5.0 have the lowest MARE and RMSPE values using the leave-one-out cross-validation, while the improved-IDW method with the maximum of seven nearest neighbors and an exponent of 5.0 has the lowest MARE and RMSPE values using the 10-fold cross-validation. It should be noted that [20] also discusses the character of the exponent and suggests that the exponent should be deduced from the form of pollution encountered. For air pollution, [20] concludes that elementary reasoning shows that the exponent should be two or three, but more sophisticated considerations could show that the exponent may vary between one and three. In our study, we use an alternative approach that the best exponent could depend on the specific outcome or measure we wanted to model. Hence, we experiment with different exponents (1.0, 1.5, 2.0, 2.5, 3.0, 3.5, 4.0, 4.5, 5.0) in order to select the one with the best performance via cross-validation and error statistics.

For a dataset with n samples, when compared with k-fold cross-validation, leave-one-out cross-validation builds *n* models from *n* samples instead of *k* models, where *n*>*k*. For leave-one-out cross-validation, each model is trained on *n* − 1 samples rather than (*k* − 1)*n/k*, as in k-fold cross-validation. Since the PM_2.5_ dataset requires a steep learning curve for the training size in question, 10-fold cross-validation can overestimate the generalization errors. The IDW-based interpolation method assumes that a point to estimate is influenced most by nearby points. Since the PM_2.5_ datasets are sparse in various locations around the contiguous United States, leave-one-out cross-validation is the better cross-validation technique for evaluating the IDW-based interpolation model, because with sparse datasets, it is better to train on as many known measurements as possible. The 10-fold cross-validation technique is best for large datasets that do not have sparse neighbors around the point to be interpolated. Because 10-fold cross-validation may leave out the best nearest neighbors in the training set, the IDW-based interpolation method will interpolate a poor estimate of the PM_2.5_ measurement. Therefore, the 10-fold cross-validation technique will show a larger error than the true error rate, as shown in the MARE and RMSPE data in Table 4.

Since leave-one-out cross-validation is the better cross-validation technique for the PM_2.5_ datasets, based on our experimental results, the best setting for the nearest neighbors and exponent are a maximum of three or four nearest neighbors and an exponent of 5.0, as shown in Table 3.

### 3.5. A Web-Based Spatiotemporal IDW Interpolation Application

A web-based spatiotemporal IDW interpolation application is designed and implemented for the improved IDW method. The system can be used to run on a server, client, through the web or from the command line. The final implementation is a web-based Java Swing application. It can be accessed at the website (www.travis-losser.com). Spatiotemporal interpolations in the large geographic area of the contiguous U.S. are CPU intensive, even after the improvements by parallel programming and the adapted k-d tree data structure. Running the application on the server would cause the server to be unresponsive when processing multiple large interpolations at once. Therefore, the system is designed to use local files from the client’s file system and to process them using the client’s CPU. A user-friendly GUI (graphic user interface) is designed for the system.

#### 3.5.1. Interpolation and Cross-Validation

**Figure 22:**
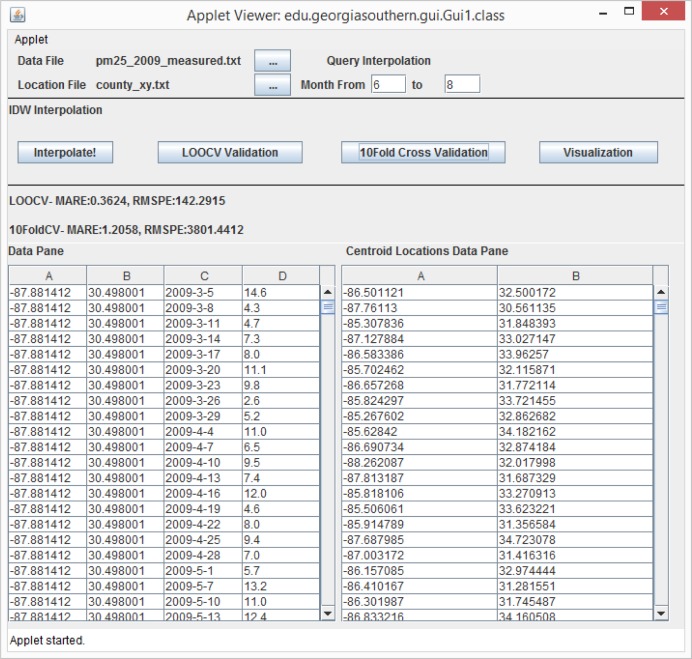
The web application GUI (graphic user interface) with a location file selected.

**Figure 23:**
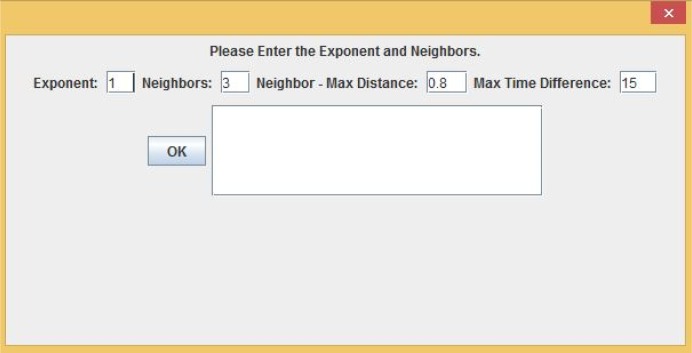
The web application GUI dialog where the user selects the exponent, the number of nearest neighbors, the maximum Euclidean distance and the maximum time difference.

There are two steps involved in performing interpolation using the web application. The first step to interpolate a dataset is to run a 10-fold cross-validation or LOOCV in order to explore a good combination of the exponent *p* and the number of neighbors *N*. Running the 10-fold cross-validation or the LOOCV function first requires the user to select a data file. If the format of the file is valid and the file is successfully loaded, the user presses the “10 Fold Cross Validation” or the “LOOCV Validation” button, as shown in Figure 22. The user is then presented with a dialog asking to select the exponent, the number of nearest neighbors, the maximum Euclidean distance and the maximum time difference, as shown in Figure 23. The maximum Euclidean distance is the maximum distance between the nearest neighbors and the point being interpolated. The maximum time difference is the maximum time difference between the nearest neighbors and the point being interpolated. Once the validation is complete, the error statistic MARE in Equation (7) and the error statistic RMSPE in Equation (8) along with some other error statistics are displayed on the GUI screen and written to a text file. The user will be responsible for choosing the optimal exponent, the number of nearest neighbors, the maximum Euclidean distance and the maximum time difference based on the error statistics results.

The second step is to click the “Interpolate!” button. A file with the locations to be interpolated needs to be selected. Once the location file is selected, the contents will be displayed in the Centroid Locations Data Pane for the user to browse, as shown in Figure 22. The Centroid Locations Data Pane displays the x and y coordinates for the centroid locations of counties or census block groups in the contiguous U.S. that are to be interpolated. When the user clicks the Interpolate button, the system will ask the user to input the exponent, the number of nearest neighbors, the maximum Euclidean distance and the maximum time difference, as shown in Figure 23, which the user should have decided from the previous validation step. Then, the interpolation is performed, and the the interpolation results are exported to a text file.

#### 3.5.2. Visualization and Animation

**Figure 24:**
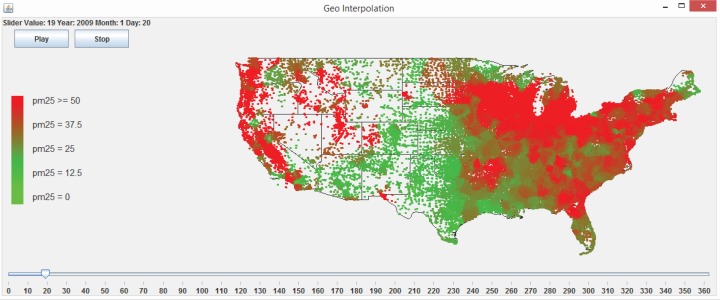
Interpolated PM_2.5_ visualization map for centroids of census block groups in the contiguous U.S. on 20 January 2009.

**Figure 25:**
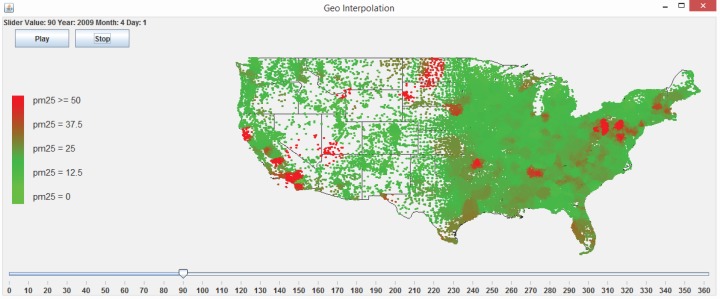
Interpolated PM_2.5_ visualization map for centroids of census block groups in the contiguous U.S. on 1 April 2009.

The “Visualization” button in the web application allows the user to visualize and animate the interpolation results for fine particulate matter PM_2.5_ overlaid on a contiguous U.S. map. The results are color rendered with the highest PM_2.5_ concentration as red and the lowest as light green. This GUI interface has two noteworthy controls. First, the bottom of the screen has a slider that can be used to select a day to view the results. As the slider is moved, the interpolated results are updated and displayed on the map. Figure 24 and Figure 25 show the PM_2.5_ interpolation results at the centroids of census block groups in the contiguous U.S. at two different time instances of 20 January 2009 and 1 April 2009. Second, clicking on the “Play” button automatically advances the time on the map and animates the daily change in PM_2.5_ over the course of the year.

## 4. Conclusions and Future Work

In order to help find associations between air pollution and health effects, this study estimates pollution levels of daily fine particulate matter PM_2.5_ for the contiguous U.S. over the year of 2009 using IDW (inverse distance weighting)-based spatiotemporal interpolation. Spatiotemporal interpolation is necessary in this study, because PM_2.5_ concentrations are measured only at certain locations and time instances by monitoring stations. IDW is one of the most popular methods of spatial interpolation. In this research, we have designed, implemented and compared two IDW-based spatiotemporal interpolation methods based on the extension approach [40] and produced daily PM_2.5_ interpolation results at both the census block group level and county level.

This study has made several contributions to the spatiotemporal interpolation research community. First, two IDW-based spatiotemporal interpolation methods have been designed, implemented and compared. One method is based on the traditional IDW method with a fixed number of nearest neighbors. Another method is an improved IDW method by excluding nearest neighbors that are far away for the interpolation point and specifying a search neighborhood. Second, when designing the IDW-based spatiotemporal interpolation using the extension approach by integrating space and time simultaneously, we calculate time values with the help of a new factor *c* in Equation (3). The assumption behind this idea is that spatial and temporal dimensions are equally important when interpolating a continuous changing data set in the space-time domain. Third, the study explores computational issues encountered when implementing spatiotemporal interpolation for large datasets and presents researchers with the appropriate techniques. Multi-threaded parallel computing techniques together with the adapted k-d tree data structure have been designed and implemented. Significant computational improvement has been achieved. The fourth contribution of this paper is concerned with finding optimal values for the number of nearest neighbors *N* and the exponent *p* for the IDW-based spatiotemporal interpolation method. The study shows how cross-validation can be used to select the appropriate *N* and *p* values. Finally, a web-based spatiotemporal interpolation application is designed and implemented. The web application has a friendly GUI and incorporates all of the work done in this study.

In future work, we would like to investigate whether there is a better way to calculate time values than using Equation (3) for the daily PM_2.5_ data. Although we have compared two IDW-based methods in this paper, we would like to further investigate different methods and compare them with the IDW-based spatiotemporal interpolation methods to see whether the interpolation performance can be further improved. Finally, we would like to link the PM_2.5_ interpolation results to various health outcomes and evaluate the PM_2.5_ pollution impact on population health.
